# Green polymeric composites based on construction and packaging waste: toward advanced insulating materials

**DOI:** 10.1038/s41598-025-22450-z

**Published:** 2025-11-04

**Authors:** Emad S. Shafik, D. A. Wissa, A. M. Labeeb, A. A. Ward, S. L. Abd-El-Messieh

**Affiliations:** 1 Polymers and Pigments Department, Chemical Industrial Research Institute, National Research Centre, Giza, Egypt; 2 Solid State Physics Department, Physics Research Institute, National Research Centre, Giza, Egypt; 3 Microwave Physics and Dielectrics Department, Physics Research Institute, National Research Centre, Giza, Egypt

**Keywords:** Waste polystyrene, Demolition waste, Sawdust, Mechanical properties, Dynamic mechanical, Engineering, Materials science

## Abstract

This study explores the development of sustainable polymeric composites using waste polystyrene (WPS) as the base matrix reinforced with sawdust (SD), red brick waste (RbW), and ceramic waste (CW). Composites were prepared with hybrid filler loadings of 0–40 wt% relative to 100 g of the total composite. Chemical composition and particle size of the solid waste were characterized by X-ray fluorescence (XRF) and Transmission electron microscope (TEM) to evaluate the probability of using these waste powders as novel alternative fillers for polymeric composites. XRF results showed high SiO_2_ and Al_2_O_3_ content in CW and RbW, while SD exhibited a high loss on ignition, confirming its organic nature. TEM results showed that the particle size of all solid inorganic wastes is in the nano size range, which makes them suitable for use as novel alternative fillers instead of traditional fillers. Mechanical tests and water absorption were measured for prepared blends, and the results revealed that tensile strength increased significantly with the addition of hybrid fillers, reaching a maximum of 25.45 MPa for the WPS/CW composite, while WPS/SD had the lowest tensile strength, which was 17.41 MPa. Elongation at break was slightly decreased with the addition of CW or RbW. Also, water absorption decreased with increasing demolition waste content, showing superior resistance to water absorption for WPS filled with CW after 15 days. Thermo gravimetric analysis, dynamic mechanical analysis (DMA), dielectric, and electrical conductivity measurements were employed to understand the thermal stability and physical properties of the composites under investigation. Dielectric measurements indicated that WPS composites filled with CW had higher permittivity and lower dielectric loss, making them recommended for electrical insulation applications. DMA also illustrated that storage modulus and glass transition temperature (Tg) enhanced with addition CW confirming enhanced thermal–mechanical stability.

## Introduction

Solid waste management (SWM) remains a critical environmental challenge, particularly in developing countries where safe and sustainable disposal methods are limited. Researchers around the world are seeking the best ways to convert waste into reusable raw materials. Governments have also been interested in encouraging waste reuse policies due to their importance from both an environmental and economic perspective. Disposing of this waste using traditional methods is expensive, including transportation expenses and the creation of sanitary landfills, as well as environmental risks such as air and soil pollution^1–5^. Additives either fillers or fibers play an important role in modifying various physical properties of polymeric composites^6–9^.

Polystyrene (PS) is one of the most important polymers used in large quantities in various industries due to its desirable properties, such as low thermal conductivity and light weight; therefore, PS foam can be used as an insulator, in lightweight protective packaging composites, and in food packaging. Furthermore, it has high compressive strength, versatility, durability, and moisture resistance^10–12^. Due to the increased use of polystyrene foam, its waste has also progressively accumulated around the world. It is considered one of the major environmental problems facing the world recently and is expected to continue in the future. Alternatively, the abandoned foam waste is non-biodegradable and resistant to photolysis. It can break down into its components, such as styrene monomers, which are categorized as potential human carcinogens, when exposed to rain, sunlight, and ocean water. For these reasons, both developing and developed countries have made numerous efforts to address this issue on a global scale^13–15^. Expanded polystyrene foam in Egypt, the problem is due to both the rapid rise in consumption and the products’ manufacturing processes. Foam waste plays an important role in reducing environmental pollution, and also these recycled materials from foam waste are not only low-cost materials used in manufacturing but also contribute to saving the utilization of products that are resultant from limited sources^16^.

Egypt is currently witnessing a tremendous development in the field of construction and slum development. Giant quantities of construction and demolition waste (CDW) have emerged alongside this development, posing an environmental and economic threat. Therefore, it has received the attention of many researchers. (CDW) presents more than one-third of all waste created in the EU. It includes a wide diversity of materials such as concrete, bricks, wood, glass, metals, and plastic. CDW contains all the waste produced by the infrastructure and buildings, as well as road development and maintenance. Waste minimization successfully requires proper waste management in all stages of development. Most of the CDW waste products cannot be reused directly and need to be reprocessed into new products^17^. Even though there has been a significant loss of natural resources, it is crucial to recognize that civil construction projects produce large amounts of construction and demolition waste (CDW) and use a significant amount of energy, which has a noticeable negative impact on the environment. In many metropolitan areas, these effects present a significant challenge, especially red brake powder and waste ceramic powder^17^.

Shafik and Youssef^18^ used porcelain tile grinding waste (PTG) as reinforced novel filler instead of kaolin as traditional filler for styrene butadiene rubber (SBR) composites. They used X-ray fluorescence and X-ray diffraction to characterize PTG. The results showed that the tensile strength, hardness shore A, and crosslinking density of SBR composites increased with increasing the PTG waste weight ratio. Dielectric measurements of SBR composites showed that permittivity increased with increasing waste content. Also, electrical conductivity σ values were found to be 10–15 S/cm, suggesting that these composites could be used for insulation purposes.

Shafik and others prepared eco-friendly composite tiles based on waste industrial gypsum mold. They prepared the composites by substituting two types of gypsum mold wastes with different ratios of raw gypsum and linear low-density polyethylene (LLDPE). The mechanical results indicated that substituting commercial gypsum with waste gypsum mold positively affected the tensile mechanical properties of LLDPE composites. Electrical tests also showed that the permittivity and dielectric loss went up as the amount of waste increased in the LLDPE composites^19^. Shafik et al. utilized red brick waste (Rbw) powder as reinforcing filler for acrylonitrile butadiene rubber (NBR) to create eco-friendly composites. Based on electrical and magnetic measurements, the conclusion strongly recommended the use of these composites as electromagnetic composites^20^. Chun and others^21^ found that the melted mixture included reprocessed polystyrene (rPS), durian shell fiber, and the treating aid Ultra-Plast TM WP516. The study presented the effects of material compositions on the morphological, thermal, and tensile behavior of the prepared composite^21^. Doha E et al.^5^ study the effect of polymer waste mix filler on polymer concrete composites. Such composites were ecologically suggested for construction requests due to the severer decrease in the carbon footprints for such samples when compared with the commercial cement. Hamdy M. Naguib et al.^6^ studied the wooden polymer composites based on polyethylene and nano-modified wooden flour with carbon nanotube which led to the conclusion that this treatment enhances its properties to be utilized as ‘‘solid-waste” for filling polyethylene.

Poletto et al.^22^ composited a product from natural fiber and WPS from WPS with wood flour. A coupling agent modified the composites, enhancing their mechanical properties and increasing their fiber loading content. Furthermore, researchers found that increasing fiber loading also led to a higher density. It can be mentioned that using EPS with wood flour is appropriate for the expansion of composite materials because of their light weight and promising mechanical properties^22^. Abdu Mohammed prepared polystyrene composites using short flax fiber that was treated with alkali as a strengthening material, and he studied how much water they absorbed and their mechanical properties^23^.

The current research aims to produce eco-friendly and economical polymer composites with desired mechanical and insulating properties based on waste polystyrene foam and hybrid fillers from sawdust and demolition waste such as red brick waste and ceramic waste. These composites may be used as wood like composites characterized by its antistatic properties. The solid wastes were analyzed using X-ray fluorescence and transmission electron microscopy to find out their chemical makeup and particle size, which helps assess if they can be used as new fillers instead of the usual ones. The research also aims to clarify the importance of using hybrid fillers, as they offer a combination of the advantages of each filler separately. Composite materials were prepared from polystyrene waste with different contents of hybrid fillers, and the mechanical and electrical properties of these composites were tested.

## Materials and techniques

### Materials

**Scheme 1:**
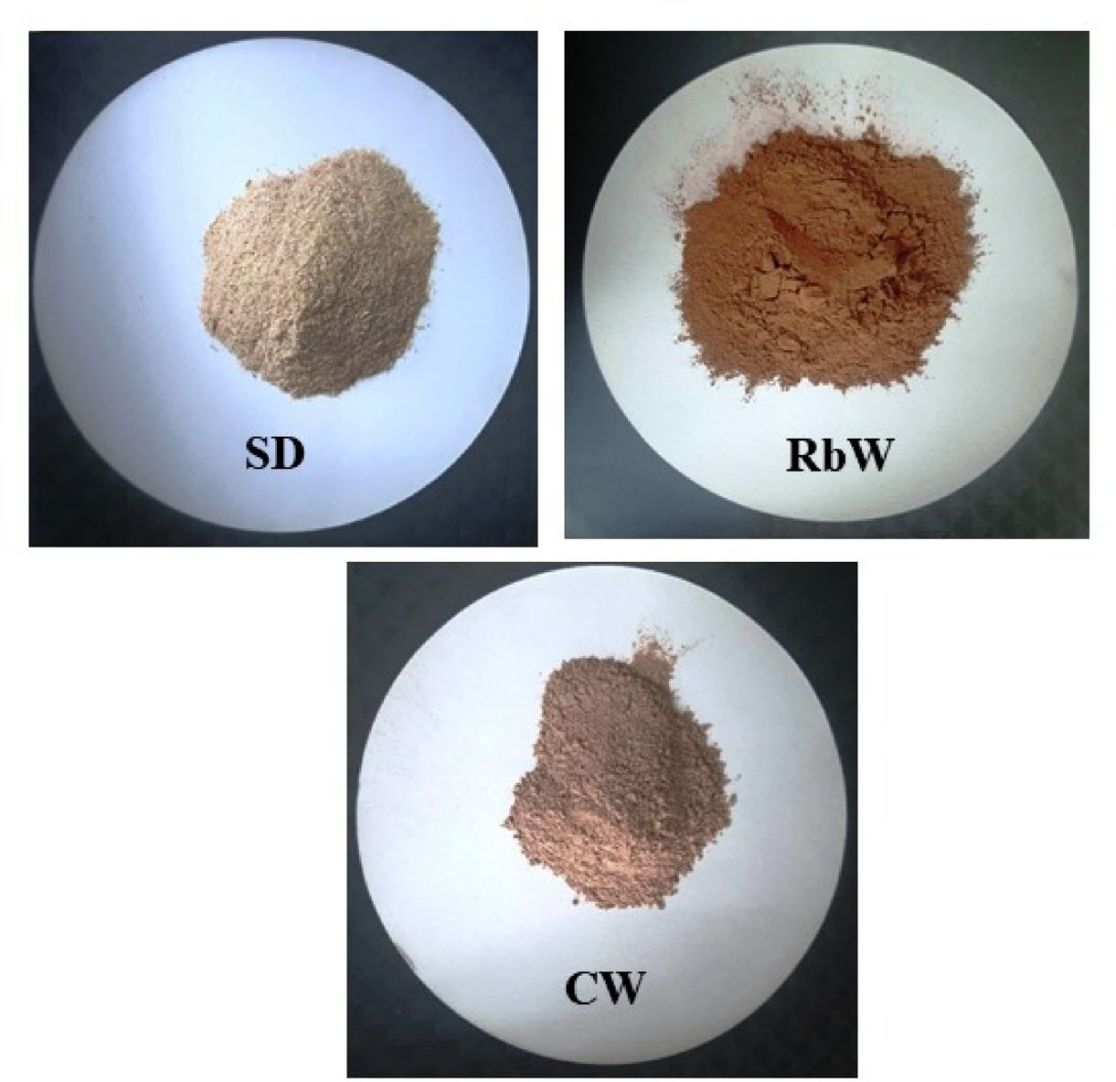
The fillers under investigations.

Waste polystyrene foam (WPS) was collected from an Egyptian plastics landfill. Sawdust was obtained from the local sawmills in Egypt. Sawdust was screened to remove the impurities and sieved through 125 µm sieve to get the same particle size. It was then dried in an oven at 103 ± 2 °C for 24 h to remove moisture before using. Red brick waste (RbW) was ground through a jaw crusher then in a mill grinder and sieved using U.S.A standard testing sieve No. 120 to get the same particle size. Ceramic waste was obtained from Egyptian factory for ceramics production as mentioned in Scheme 1.

### Assessment and characterization of waste

All waste powders were analyzed by X-ray fluorescence technique (XRF), Type: Axios, Sequential mod. (WD-XRF), PA Nalytical 2005 obtained from Netherlands, and the particle size of waste powders were also examined by Transmission Electron Microscopy (TEM) JEOL JX 1230.

### Density measurements

A sample’s density is calculated from its weight in air and its apparent weight when immersed in a liquid of known density, using Archimedes’ Principle. The procedure was carried out as the following, in first, the setup which was started by mounting the Archimedes kit on the Bel analytical 5-digits balance. Attach the sample holder and tare. Second, weigh in air (W_l_) was performed by placing the dry sample in the holder and record its weight. Third, weigh in liquid (Absolute ethanol at 25 °C (Wₗ), Submerge the sample fully in the liquid. Ensure no bubbles then recording the apparent weight (often negative). Keeping in mind the density (ρ_l_) the density of the liquid at the current temperature is 0.784 g/cm^3^.The equations are used in the calculation of the density is as the following is according to ASTM-D7481-09:

$${\rho }_{s}=\frac{{W}_{a}.{\rho }_{l}}{{W}_{a}-{W}_{l}}$$

where ρ_s_ is sample density (g/cm^3^), W_a_ is weight in air (g), W_l_ is weight in liquid (g) (use the negative value as recorded), and ρ_l_ is liquid density (g/cm^3^). The experiment was repeated three times to determine the relative and precision errors.

### Preparation wood plastic composites

Waste Polystyrene (WPS) was mixed in an internal mixer (Brabender Plasticorder) at 30 rpm and temperature 170 °C. First, WPS was included to the mixer, and the different filler powders with different content were included following the reaching of the polymer melting temperature. After 5 min in average, the resultant composites was removed and pressed into different dimensions using laboratory hydraulic hot press at 170 °C for 5 min and then cooled at room temperature for about 10 min.The formulation of the samples was listed in Table 1.

**Table 1:** Formulations of wood plastic composites.

Ingredients, g	Blank WPS	WPS/SD/RbW composites	WPS/SD/CW composites
Waste polystyrene WPS	100	60	60
Sawdust SD	–	40, 30, 20, 10, 0	40, 30, 20, 10, 0
Red brick waste RbW	–	0, 10, 20, 30, 40	–
Ceramic waste CW	–	–	0, 10, 20, 30, 40

### Characterization of wood plastic composites

#### Mechanical and water absorption properties of prepared composites

Mechanical properties of prepared composites including tensile strength and elongation at break were measured according to ASTM D638 using an electronic Zwick tensile testing machine (model Z010, Germany). Also, hardness shore D was evaluated according to ASTM D2240, using Hardness tester Model HR-150A Rockwell. Water absorption was conducted for prepared composites according to the ASTM D 570 water absorption ratios were also measured and calculated according to the following equation.

$$Water absorption \%= \frac{Ww-Wd}{Wd} X 100$$

where W_w_: Weight of wet sample, W_d_: Weight of dry samples.

Each measurement was the mean of four samples and the experimental error was within 3%

#### Thermal and structural morphology of prepared composites

Thermo gravimetric analysis (TGA) was carried out via a TGA instrument, the TGA/DSC 3 + (Mettler Toledo Inc, USA), with nitrogen flow from 30 to 600 °C and a heating rate of 10 °C per minute. Additionally, a scanning electron microscope was used to observe the morphology of the produced composites (SEM, JSM 7600F, JEOL, Tokyo, Japan).

#### Dielectric and conductivity measurements

The Schlumberger Impedance/Gain-Phase Analyzer 1260, functioning within a frequency range of 0.1 Hz to 1 MHz, was employed to assess the permittivity ε′, dielectric loss ε″, and alternating resistance R_ac_ at ambient temperature (30 ± 1 °C). Three samples are utilized for each measurement, and the average is thereafter computed. The measurement error in ε′ and tan δ is ± 1% and ± 3%, as well. The samples’ temperature was regulated by a temperature controller utilizing a Pt 100 sensor. The measurement error in temperature measurements is ± 0.5 °C.

#### Dynamic mechanical properties

The storage modulus (E′), damping factor (tan δ), and glass transition temperature (T_g_) of the examined materials were determined using a dynamic mechanical analyzer (DMA1, Mettler Toledo, Switzerland). Measurements were conducted in dual cantilever bending mode using ASTM D4065, which specifies techniques for the dynamic mechanical property evaluation of polymers. Specimens underwent cyclic tensile strain at a fixed frequency of 1 Hz and a force amplitude of 0.1 N. The storage modulus (E′) and mechanical damping factor (tan δ) were measured across a temperature spectrum of 30–150 °C, with a heating rate of 5 °C/min.

## Results and discussion

### Characterization of waste polystyrene and waste powders

X-Ray fluorescence with the weight percent for each element for all waste powders were illustrated in Table 2. The main contents that characterize RbW was the presence of SiO_2_ (53.55), Al_2_O_3_ (17.83) and Fe_2_O_3_ (8.88) while those for CW was SiO_2_ (62.81), Al_2_O_3_ (19.45) and considerably low amount of Fe_2_O_3_ (3.06). In case of SD the main content was the loss on ignition LOI as it consists mainly of wood. The obtained results explain the good magnetic properties of the composites that contain RbW rather than that contain CW which have not any magnetic response due to the low amount of Fe_2_O_3_.

**Table 2:** XRF analysis of three waste powders.

Main constituents	Wt%		
	RbW	CW	SD
SiO_2_	53.55	62.81	0.09
TiO_2_	0.97	0.57	0.06
Al_2_O_3_	17.83	19.46	0.03
Fe_2_O_3_	8.88	3.06	0.10
MgO	2.08	1.44	0.05
CaO	3.25	2.34	0.34
Na_2_O	2.09	2.28	0.04
K_2_O	0.99	0.86	0.06
SO_3_	5.69	0.39	0.05
P_2_O_5_	0.24	0.37	0.02
Cl	0.30	0.15	0.16
LOI	3.70	5.22	98.99

### Density measurements

Table 3 clear the density of fillers, Brick dust consists of fine particles with air gaps, so its bulk density is lower. Literature values for the bulk density of brick dust (or brick powder) vary bulk density of brick dust is often in the range of 0.4–0.8 g/cm^3^, depending on particle size and compaction. For example, finely ground brick dust might have a bulk density around 0.5–0.6 g/cm^3^.Some sources report values as low as 0.4 g/cm^3^ for loose brick powder. Our calculated value (0.4635 g/cm^3^) falls within the typical range for loose brick dust. It is slightly on the lower end, which might be due to the sample being very fine and having high porosity^24^.

**Table 3:** Density of the fillers under investigations.

Materials	Density, ${\rho }_{s}$, (g/cm^3^)	Standard deviation, (± g/cm^3^)
RbW	0.4635	0.0022
CW	0.3808	0.0034
SD	0.1522	0.0051

Bulk density of ceramic powder, which the density value of ~ 0.381 g/cm^3^ is perfectly reasonable for the bulk density of a fine, loose powder. Bulk density includes the air trapped between the particles. A low bulk density indicates a very fine powder that packs loosely with a high volume of air voids. This value is consistent with other fine, mineral-based powders like brick dust or certain types of fly ash. A study on using waste ceramic powder as a filler in composites might report its bulk density. For example, research articles often report loose bulk densities of ceramic powders in the range of 0.3–0.7 g/cm^3^, depending heavily on particle size distribution and morphology^25^. The density of SD can vary depending on the wood type, but typically it is around 0.2–0.5 g/cm^3^ for many common woods. However, saw dust might be less dense due to air trapped between particles. Our calculated value (0.1522 g/cm^3^) is on the lower end, which might be due to the high porosity and the fact that saw dust particles have air gaps. Literature values for bulk saw dust density are often in the range of 0.1–0.3 g/cm^3^, so 0.1522 is reasonable^26^.

### TEM measurements

**Fig. 1:**
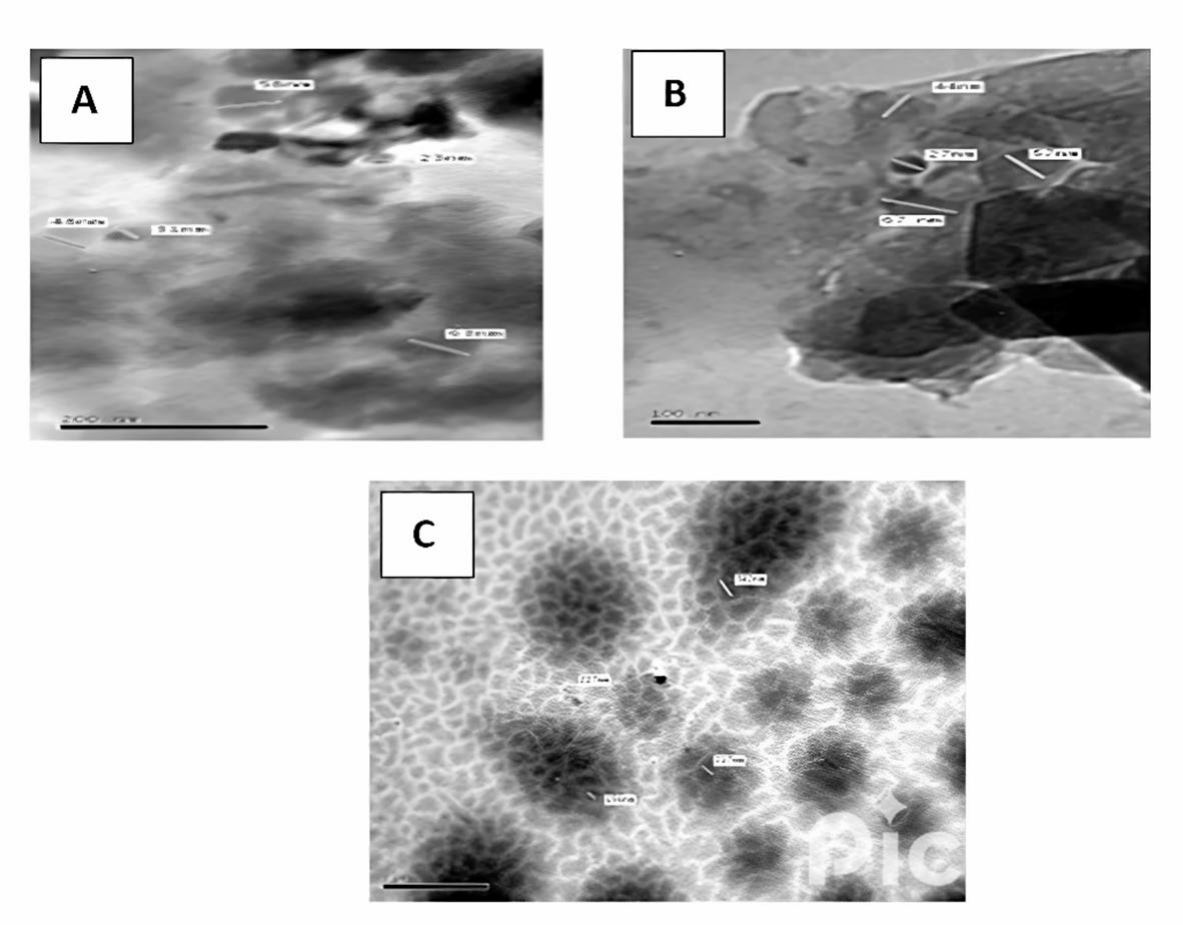
TEM images for Fillers under investigation. (**a**) RbW, (**b**) CW, (**c**) SD.

The TEM was used to determine the particle size of the used fillers and the obtained images were given in Fig. 1. From this figure it is seen that the average particle size was about 45 nm for RbW, 49 nm for CW and 263 nm for SD respectively.

### Characterization of prepared composites

#### Mechanical and water absorption of prepared composites

**Fig. 2:**
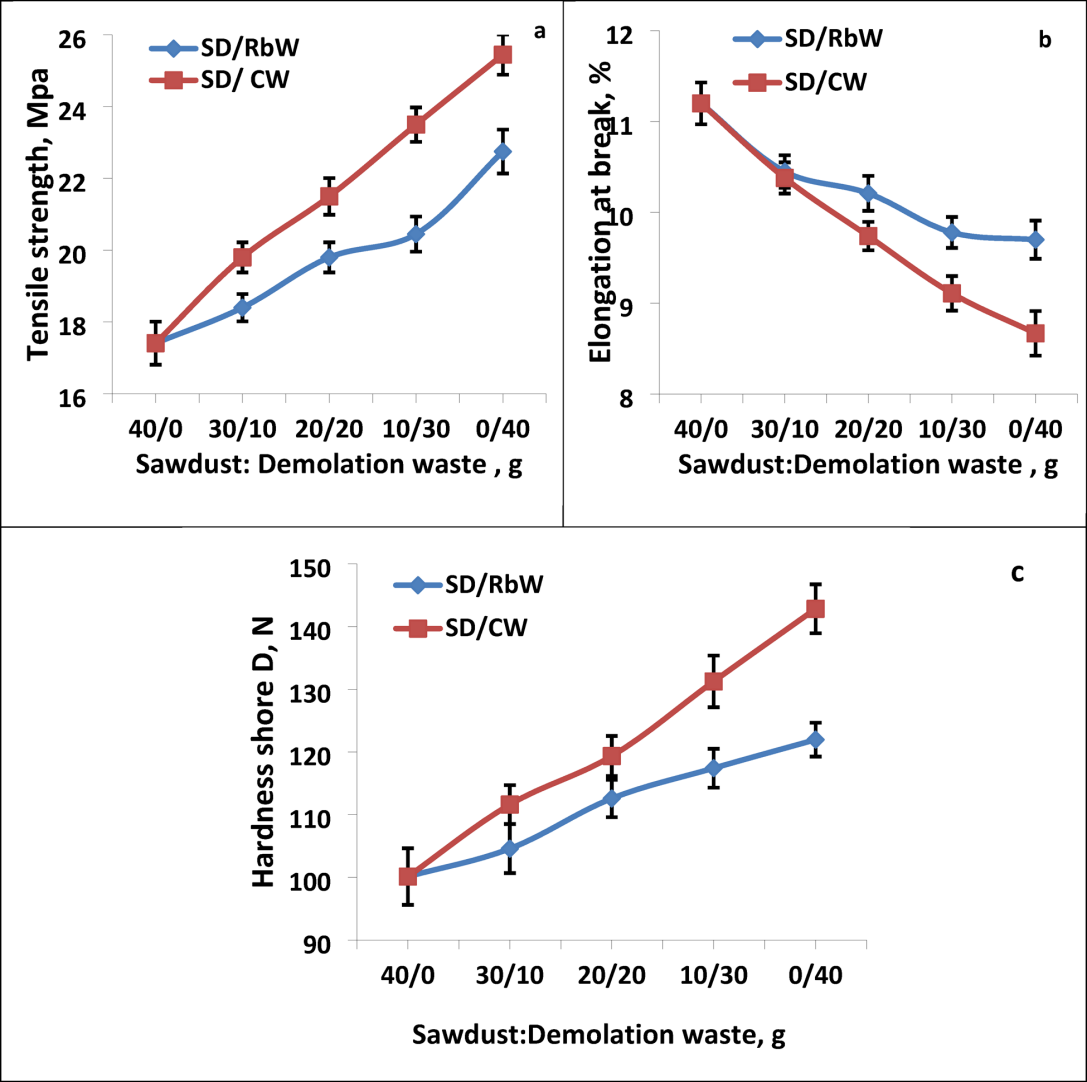
Mechanical properties of waste polystyrene composite filled with hybrid filler from sawdust and demolition waste. (**a**) Tensile strength, MPa, (**b**) Elongation at break %, and (**c**) Hardness Shore D,N.

Hybrid fillers are one of the most important new fillers currently used in many industries. They utilize the advantages of each component in a single composite. Therefore, two types of hybrid fillers were used. The first type is a mixture of sawdust and red brick powder at various replacement content, as shown in Table 1. The second type is a mixture of sawdust and ceramic waste. Figure 2a–c shows the mechanical properties of waste polystyrene composites filled with two previous types of hybrid fillers. From Fig. 2a, it is clear that the waste polystyrene composite filled with sawdust achieved the lowest tensile strength, which was 17.41 MPa. A gradual increase in tensile strength was also observed with the use of hybrid fillers, especially SD/CW more than SD/RbW, until the maximum tensile strength reached 25.45 MPa for the polystyrene composite containing 100% CW. The increase in tensile strength may be due to the strong interfacial adhesion between the polymers chain and waste fillers, which improves load transfer efficiency^27^. Figure 2b represents the elongation at break of the polystyrene waste composites. From the figure it is clear that the waste polystyrene filled with sawdust filler achieved the highest elongation value, which was 11.2%. It is also clear that there was a slight decrease after adding hybrid fillers to the polystyrene waste, until it reached its lowest values of 9.7 and 8.67 for the polystyrene waste containing 100% RbW and CW, respectively. The elongation at break reduction is due to the RbW and CW increase the stiffness of the composites under test^20^. Figure 2c also represents the hardness of the composites. The figure shows that the hardness gradually increased, from 100.13 Newtons for the polystyrene waste filled with sawdust fillers, to its highest values of 121 and 142 for the polystyrene composites containing 100% red brick waste and ceramic waste, respectively.

**Fig. 3:**
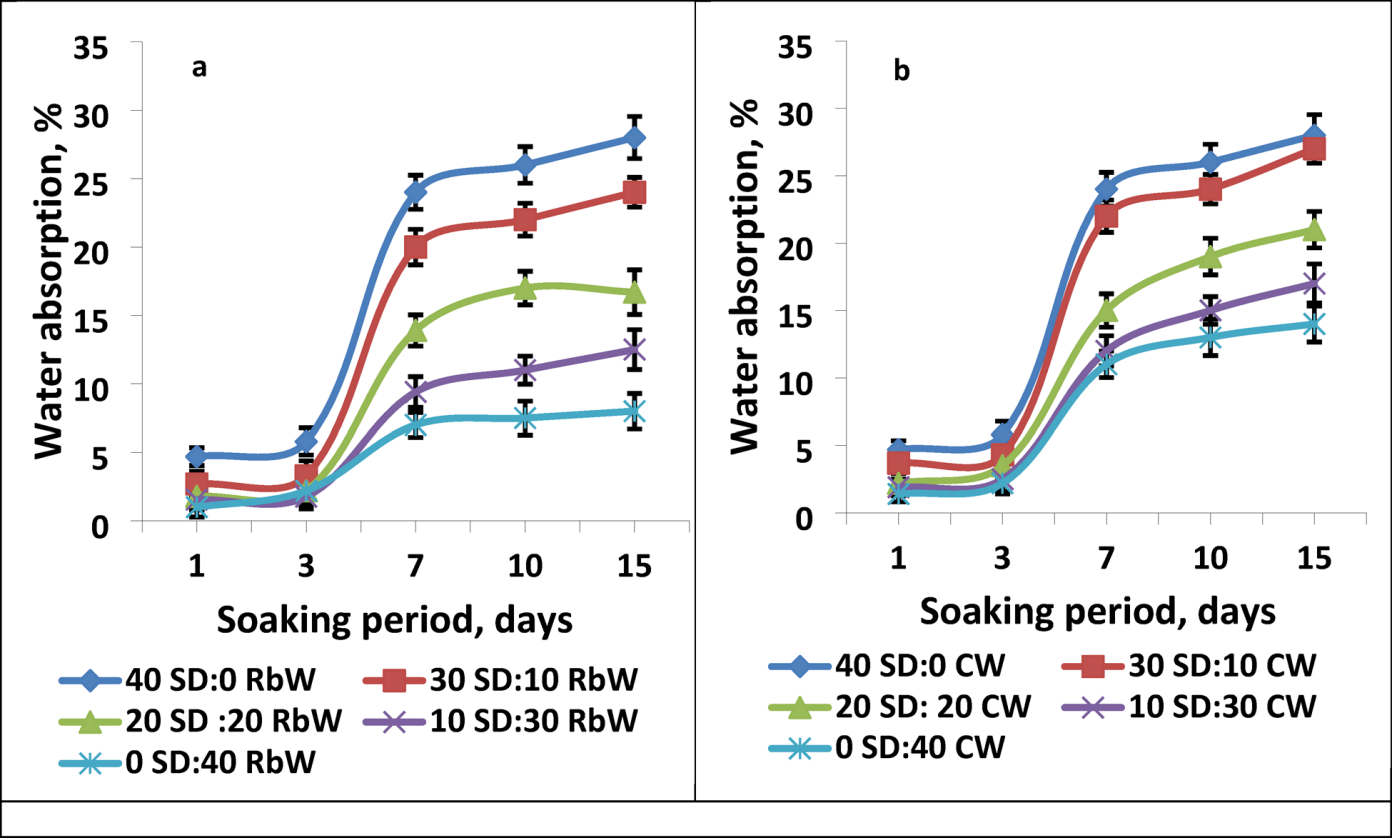
Water absorption ratio of waste polystyrene composites filled with hybrid fillers from sawdust and demolition waste.

Figure 3 shows the relationship between water absorption and different soaking periods up to 15 days for waste polystyrene composites filled with hybrid fillers from sawdust and demolition waste such as RbW and CW. Figure 3a shows that the water absorption ratio for all composites gradually increases with increasing soaking period. It is also clear that with increasing RbW content, the water absorption ratio decreases. The water absorption ratio was at its highest, reaching 28% after 15 days for the waste polystyrene composite filled with sawdust only, and gradually decreased in the presence of RbW to 8% for the waste polystyrene composite containing RbW only. Figure 3b also shows that the waste polystyrene composite filled with CW achieved the lowest water absorption ratio compared to sawdust only, with an absorption ratio of 14%. This finding is logic because of the ability of SD for absorbing water. Also it was find that the samples contain CW is more resistance to water absorption rather that those contain RbW.

#### Thermal and structural morphology of prepared composites

**Fig. 4:**
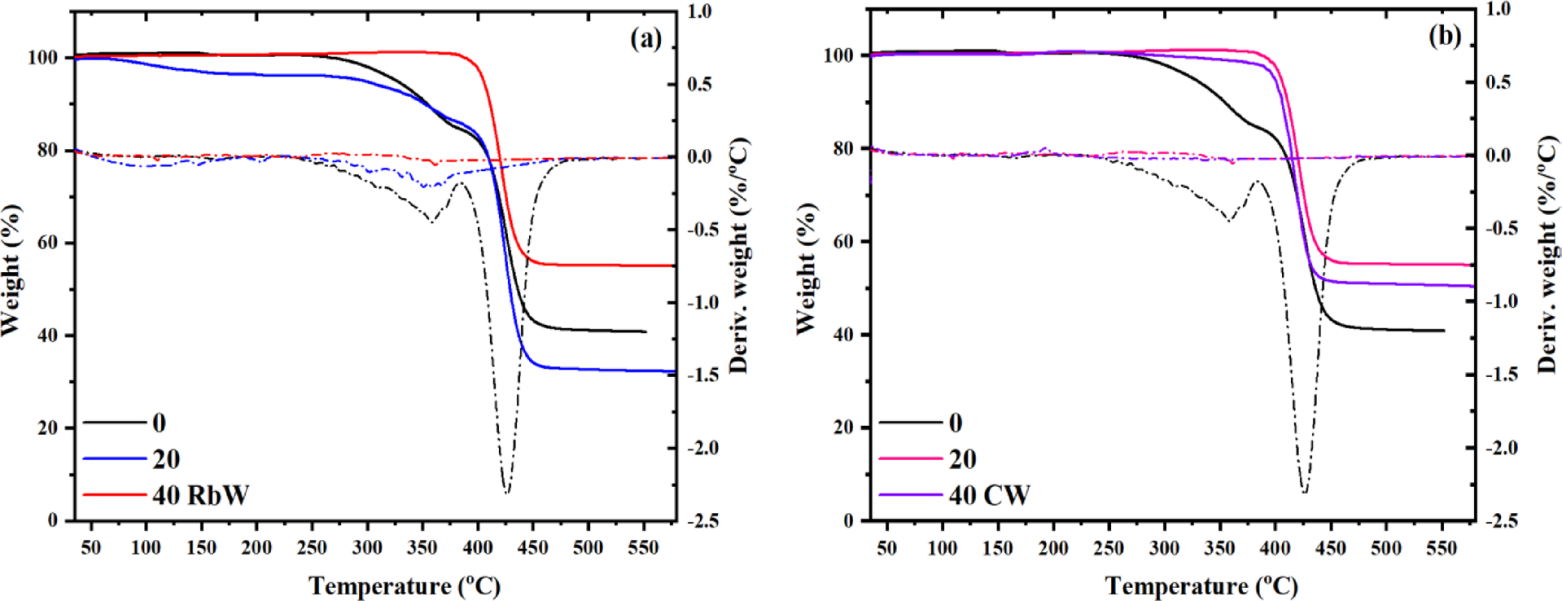
TGA thermographs for WPS composites filled with sawdust and demolition waste.

Figure 4a, b depicts the thermogravimetric (TGA) and derivative thermogravimetric (DTG) characteristics of WPS composites including sawdust (SD) and hybrid fillers sourced from demolition waste, namely red brick waste (Rb) and concrete waste (CW). The TGA curves indicate a slight initial weight reduction below 150 °C in composites including SD and 20 g of either Rb or CW. This initial thermal occurrence is probably linked to moisture evaporation and the emission of low-volatility organics, in accordance with the elevated organic content of SD (LOI: 98.99%, Table 2). The estimated onset temperatures for these samples range from 91 to 144 °C.

In contrast, composites comprising mainly RbW or CW demonstrate no loss of weight in this low temperature range, indicating enhanced thermal stability due to the primarily inorganic composition of the fillers. This difference is further clarified by the DTG plots, which show the absence of a corresponding peak in the low-temperature range for composites containing RbW and CW, confirming minimal decomposition activity. A second significant deterioration event occurs between 250 and 400 °C in all samples. The DTG curves exhibit significant peaks in this region, indicating the major degradation of the polymer matrix. The strength of these DTG peaks notably decreases with more RbW or CW content, indicating a delay in thermal deterioration and improved char formation. This result demonstrates the stabilizing influence of inorganic fillers, which probably act as heat insulators and enhance residue retention.

The TGA and DTG analyses together indicate that the inclusion of RbW or CW in WPS composites enhances thermal resistance at both initial and elevated temperature stages, with DTG offering a more precise understanding of the kinetics and extent of breakdown transitions.

**Fig. 5:**
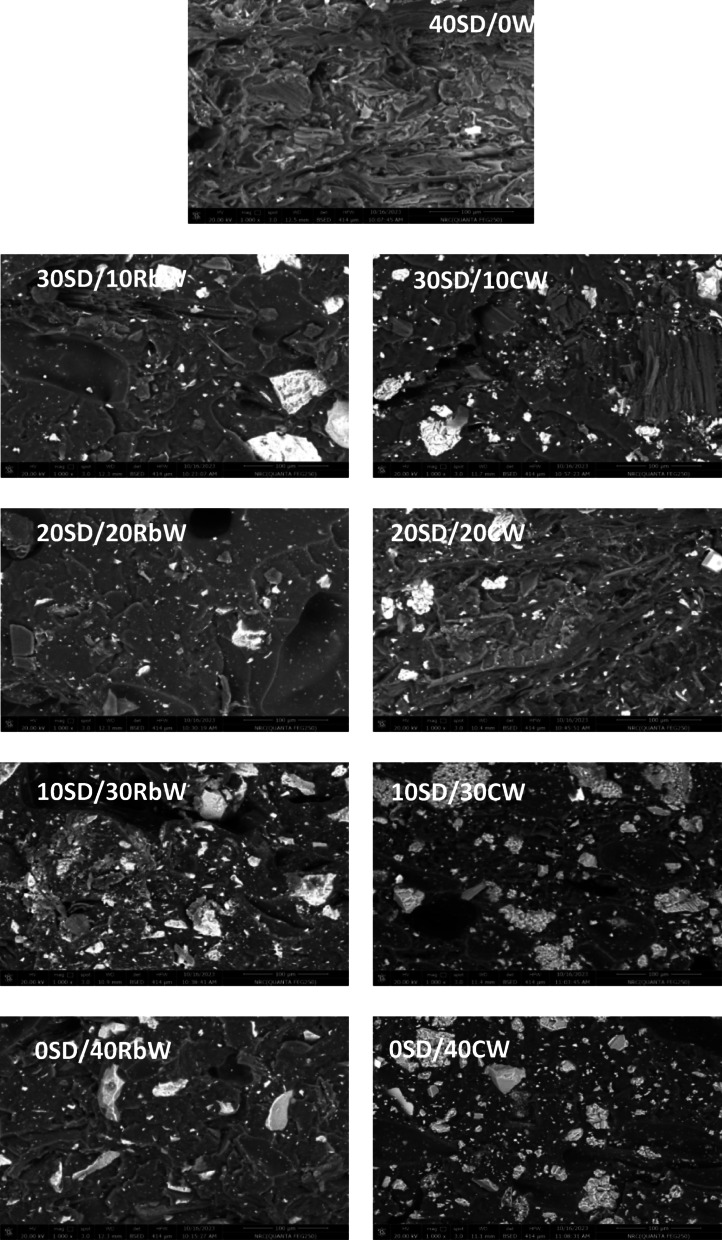
Scanning electron microscopy of Waste polystyrene composites.

The SEM is a tool that shows the morphology of the polymeric composites. During the “scanning” procedure, the electron beam interrelates with the surface aerie and produces secondary electrons from the sample. Scanning electron micrograph was measured at magnification of 1000 for the two series WPS/SD/RbW and WPS/SD/CW composites and the obtained micrographs were listed in Fig. 5. These micrographs reveal homogenous distribution of the hybrid filler only when the ratio of SD with either RbW or CW was 20/20 while on the other hand, the other content show aggregations of filler inside the WPS matrix.

#### Dielectric measurements

**Fig. 6:**
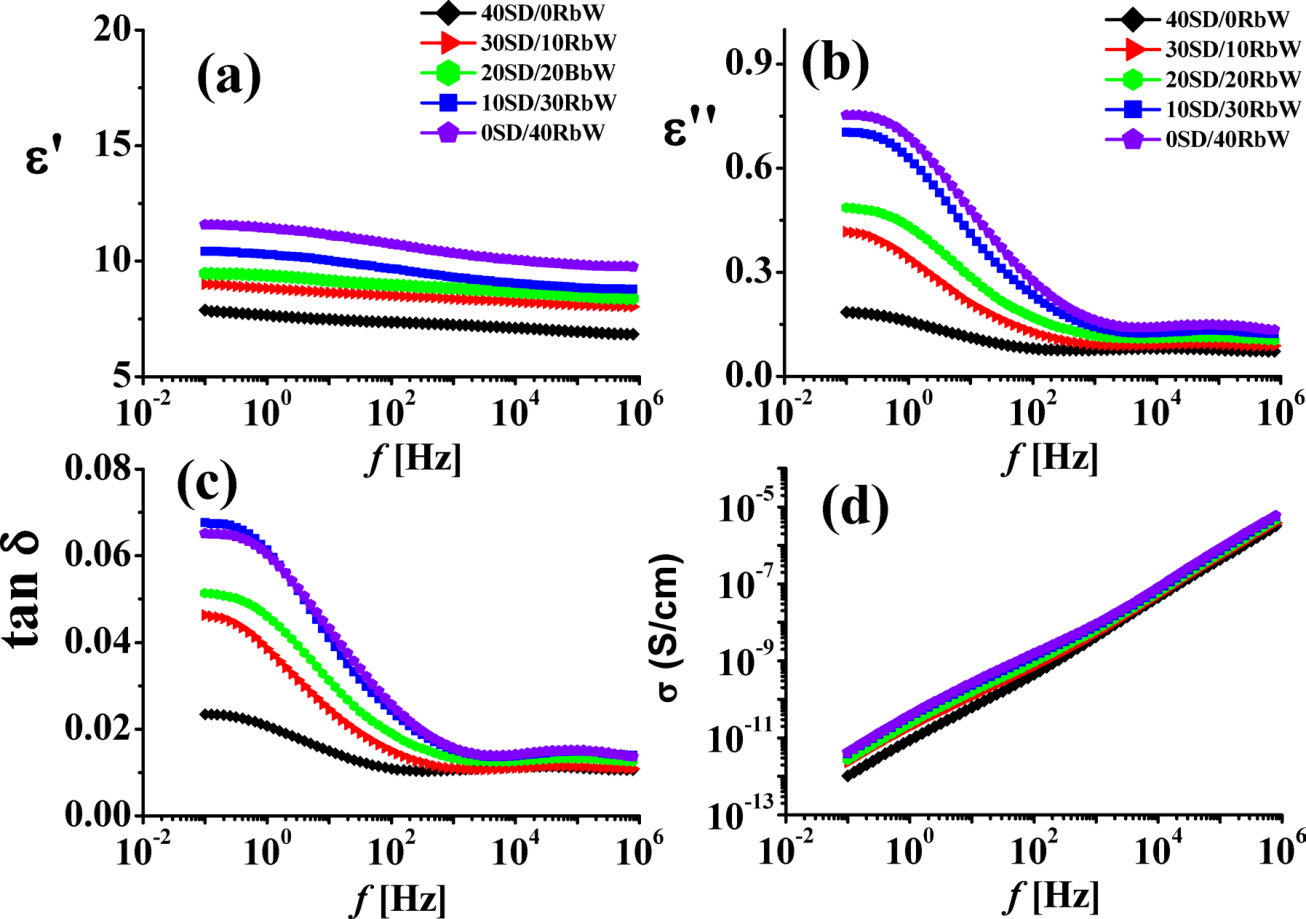
Permittivity ε′, dielectric loss ε″, loss tangent tanδ and conductivity σ as functions of frequency *f* for WPS composites filled with different content of Sawdust/Redbrick (SD/Rbw) fine powder.

**Fig. 7:**
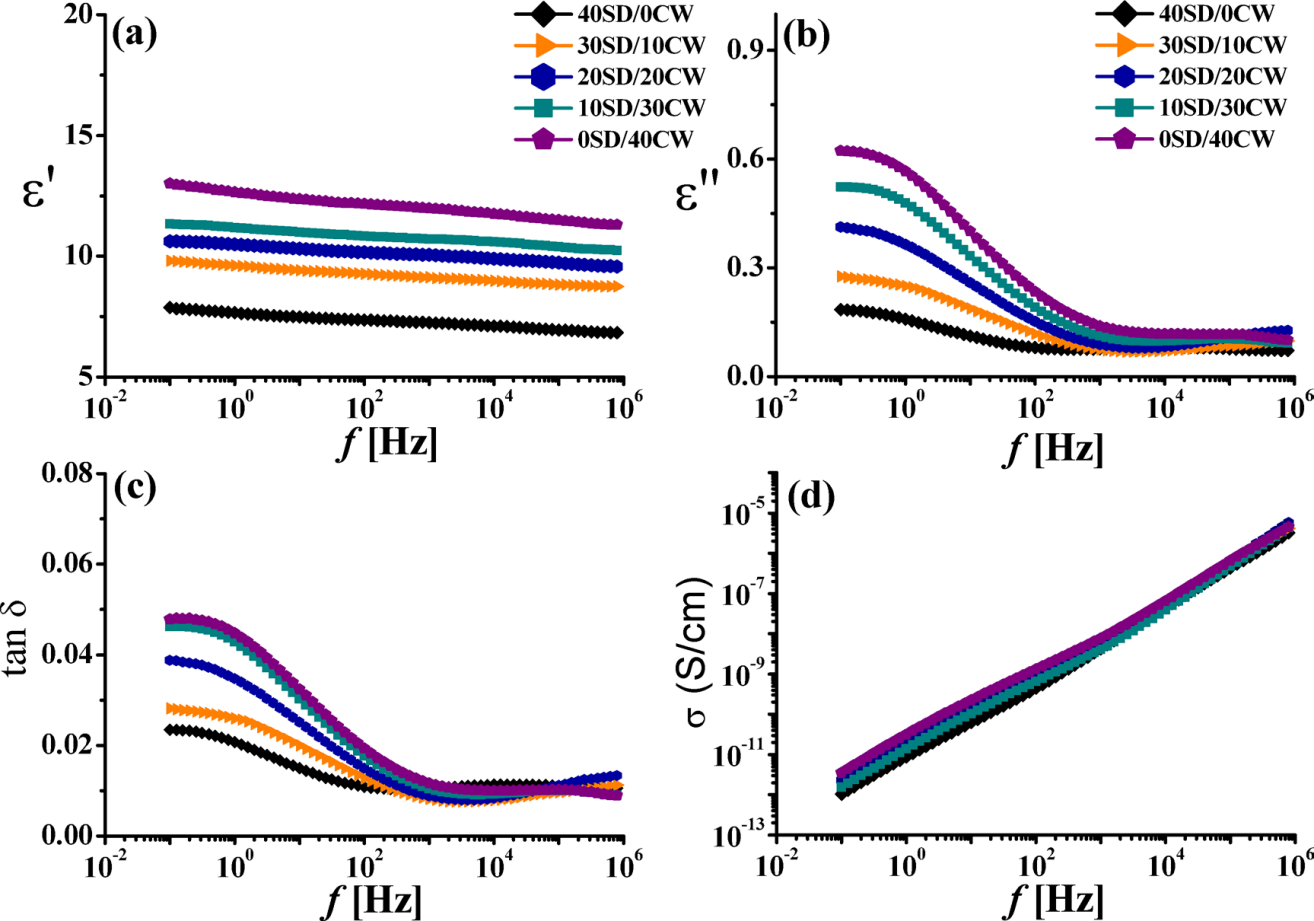
Permittivity ε′, dielectric loss ε″, loss tangent tanδ and conductivity σ as functions of frequency f, for WPS composites filled with different content of SD/CW fine powder.

The dielectric parameters including permittivity (ε′), dielectric loss (ε″), loss tangent (tanδ) and AC electrical conductivity (σ) were measured at room temperature 25 °C for the two series under investigation and the obtained data were given in Figs. 6, 7 respectively.

Many factors influence of the ε′ composites such as filler dispersion and filler morphology in addition to filler matrix interaction^28^. By increasing f, slight decrease in ε′ was remarked. This implies the rotational motion of dielectric polar molecules is not fast enough to achieve balance with the field^29^. Dielectric relaxation phenomena shows that interface polarization is a significant contributor to ε′ of the composites this behavior, which is typical of most polymer dielectrics and is brought on by the polymer materials.

**Fig. 8:**
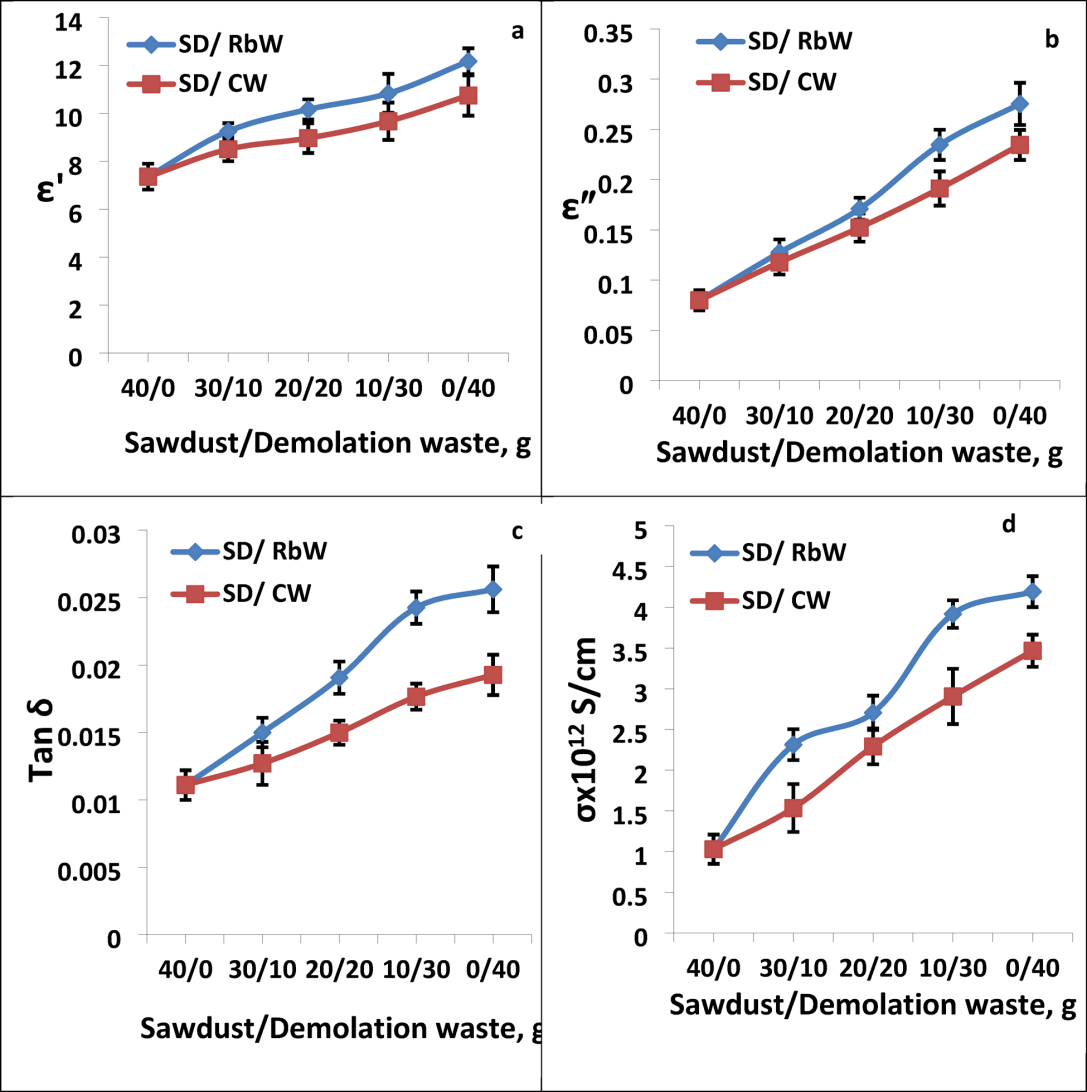
The dielectric parameters, loss tangent tanδ and conductivity versus hybrid filler content at fixed f = 100Hz at room temp. 25°C.

The addition of filler particles increases ε′ of the composites all over the f, range. At higher f, the rotating movement of the molecules lags after the electric field, causing in decrease in ε′ as f, increases. On the other hand, with regard to both ε″ and tanδ, both values first exhibits a rapidly decreasing trend in the low frequency phase before progressively slowing down in the high frequency phase, as seen in Figs. 6, 7. In contrast, the absorption curves of ε" VS. *f* shown in Figs. 6,7 are complicated, point out the existence of extra than one relaxation procedure in addition to dc conductivity σ^30–33^. In order to follow-up the changes happened in both ε′, ε" and tan δ of the composites both values were depicted in Fig. 8 as a function of filler content at 100 Hz and temp. = 25 °C. It is clear that all dielectric parameters increase by increasing both RbW and CW content in the composite.

Also, it is seen from Fig. 8a that ε’ values are slightly high for composites contain CW than those contain RbW, this may be due to the high percentage of both SiO_2_ and Al_2_O_3_ in CW, see Table 2. On the other hand, both ε” and tanδ as shown in Fig. 8b, c values are the contrary. This finding recommends CW composites to be used as electrically insulator composites. The impact of various effective of AC conductivityσ versus f is depicted in Figs. 6, 7 where it is reflects that the composites conductivity increase quickly as f increase. The compositesσ increases as a result of electric dipole inside it became more active and the charge be in motion is more force full at this time. It is well-known that, the total f, reliant conductivity, σ (ω) is a sum of dc and ac constituents as explained elsewhere^34^.

The dc σ was calculated from the extrapolation of the ac σ at zero f, and the gotten data are listed in Fig. 8d. Based on this Figure it is found that σ dc increase by increasing filler content either RbW or CW but it was slightly higher in case of CW. The applications of the polymer composites depend mainly upon the value of its electrical conductivity^35^. For anti-static relevance the range of σ was 10^–9^–10^–14^/S/cm while for electrostatic dissipation relevance the range of it is 10^–5^–10^−9^S/cm. For semi-conducting materials, used in power cables to stop partial discharge at the boundary between insulation and conductor, the demanded σ was in the range of 10^−3^S/cm^36–38^. From Fig. 8d it was found that the σ_dc_ value was in the range of 10^−12^S/cm that recommends such composites for anti-static relevance. This finding supports the presumption of using such composites as wood like ones.

#### Dynamic mechanical analysis (DMA)

**Fig. 9:**
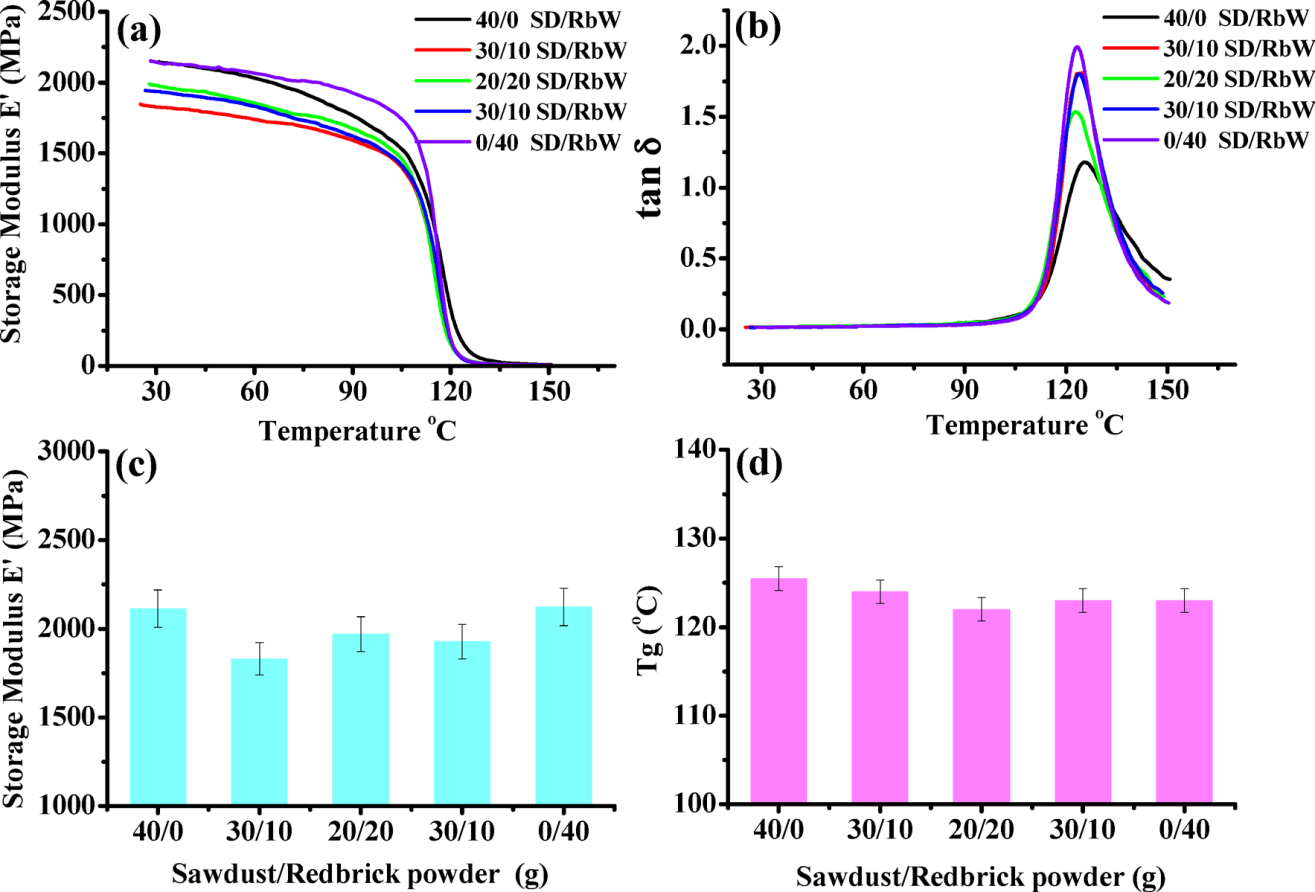
Storage modulus E’ and loss factor tan δ as functions of temperature (30–150 °C) of WPS composites filled with different content of SD/RbW powder.

The samples were mounted in the Single Cantilever Bending clamps and run from ambient to 150 °C. Figure 9a, b demonstrates the storage modulus (*E'*) and damping (tanδ) of the waste styrene composites filled with different content of SD/RbW as functions of temperature (30–150 °C). The storage modulus (*E'*) in Fig. 9a, c of sample free from RbW is 2143 MPa drops to 1831, 1967, 1928 MPa by incorporating 10–30 g redbrick powder then rises to 2123 MPa. On the other hand, the decrease of E*'* with temperature characterizes the “transition zone” denoted to as “glassy region” where there are limitations on segmental mobility and molecular motions. However, a rubbery area where the modulus and damping values drastically decrease as the temperature rises until they reach constant values. Moreover, Fig. 9c, d, presents the obtained results of storage modulus* E*′ at 30 °C and glass transition temp. T_g_ calculated from the peak maximum of tan δ Fig. 9b. In this figure, T_g_ decreased by 2°–3° by incorporation of redbrick waste. Clearly, the reduction of T_g_ for the waste polystyrene/sawdust composite redbrick powder points to the slight break of interchange bonding, which resulted in a more flexible backbone.

**Fig. 10:**
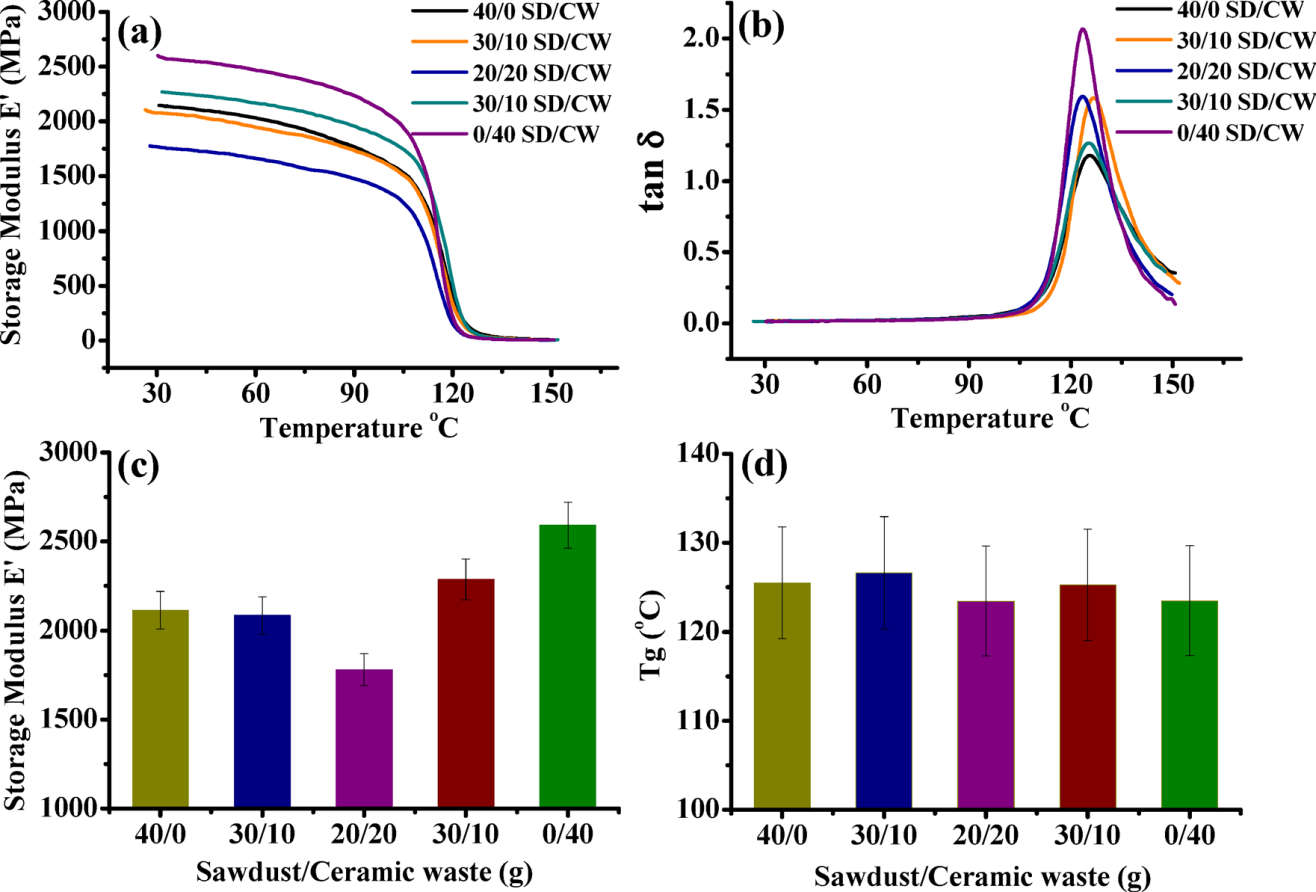
Storage modulus E’ and loss factor tan δ as functions of temperature (30–150 °C) of WPS composites filled with different content of SD/CW powder.

The samples were mounted in the Single Cantilever Bending clamps and run from ambient to 150 °C. Figure 10a, b demonstrates the storage modulus (*E'*) and damping (tanδ) of the waste styrene composites filled with different contents of sawdust/ceramic fine powder as functions of temperature (30–150 °C). The storage modulus (*E'*) in Fig. 10a, c of WPS composite containing SD only is 2143 MPa slightly decreased to 2085, and then drops to drops to 1780 MPa. E′ rises to 2287 and 2592 MPa by incorporating 30, 40 g ceramic waste powder. Alternatively, the decrease of E’ with temperature characterizes the “transition zone” denoted to as “glassy region” where there are limitations on segmental mobility and molecular motions. However, a rubbery area that exhibits a sharp decline in damping and modulus values as the temperature rises to constant values. Moreover, Fig. 10c, d, presents the values of storage modulus *E’ at* 30 °C & glass transition temp. T_g_ calculated from the peak maximum of tan δ Fig. 10b.

In Fig. 10c, d it is clear that, T_g_ rises after adding 10 g ceramic powder. This may be due to the reduction of mobility and/or flexibility of the polymer chains as a result of the polymer’s adsorption on the filler at the interface, T_g_ shifts towards a higher temperature as a result of this drop. While, the decrease observed in Fig. 10d by increasing CW powder content, indicates that the interchange connection was slightly broken, resulting in a more flexible backbone.

## Conclusion

In this study the potential of the incorporation of hybrid filler from sawdust and demolition waste including RbW and CW with various content as reinforcing fillers for WPS aiming to obtain composites totally coming from waste to be used as artificial wood composites. Filler characterization led to the conclusion that the main contents that characterize RbW which has particle size about 45 nm was the presence of SiO_2_ (53.55), Al_2_O_3_ (17.83) and Fe_2_O_3_ (8.88) while those for CW with particle size about 49 nm was SiO_2_ (62.81), Al_2_O_3_ (19.45) and considerably low amount of Fe_2_O_3_ (3.06). In case of SD, which has the largest particle size about 236 nm, the main content was the loss on ignition LOI as it consists mainly of wood. The samples micrographs reveal homogenous distribution of the hybrid filler only when the ratio of SD with either RbW or CW was 20/20 while on the other hand, the other ratios show aggregations of filler inside the WPS matrix. The mechanical investigations reveal that the increase in the filler loading is accompanied with marked increase in the tensile strength and hardness which indicate the reinforcement effect of the filler. On the other hand, pronounced decrease in the elongation at break was noticed for both composites. From the water absorption test it was concluded that the samples contain CW is more resistance to water absorption rather that those contain RbW. From the dielectric studies it was concluded that ε′ values are slightly high for composites contain CW than those contain RbW, while both ε″ and tanδ values are the contrary. This finding recommends CW composites to be used as electrically insulator composites. The σ_dc_ values for all composites were in the range of 10^–12^ S/cm that recommend such composites for anti-static relevance. This finding supports the presumption of using such composites as wood like ones. DMA study examines the impact of adding sawdust/redbrick and ceramic fine powder on the storage modulus (E') and damping (tanδ) of waste styrene composites from 30 to 150 °C. The incorporation of redbrick powder results in slight variation in E’ values compared to the blank sample. In addition, a slight decrease encountered in glass transition temperature (T_g_) by 2°–3°. This decrease arises from minor disruption in interchange bonding, leading to a more flexible backbone. On the other hand, the addition of ceramic powder shows a similar trend, with a rise in E’ and T_g_ due to reduced polymer chain mobility.

## Data Availability

The datasets used and/or analyzed during the current study available from the corresponding author on reasonable request.
